# Clinical Audit of Transfusion Safety and Monitoring at Al-Kareemat Specialized Complex, Sudan: A Two-Cycle Quality Improvement Study

**DOI:** 10.7759/cureus.102769

**Published:** 2026-02-01

**Authors:** Shimaa Ali Hassan Hamed, Abubaker Ibrahim Mohammed Ibrahim, Zainab Abdelrahman Mohamed Ali, Ahd A Ahmed Abdelghani, Rowida A Ahmed, Sana Taha Ibrahim Taha, Yosria Alneel Edrees Alneel, Sara Hassan Siddig Mohamed, Rana Mohamedosman Babiker Karrar, Amna Alhafiz, Asim Ahmed, Nosiba Abd Alraheem Aljack Awad Alkareem

**Affiliations:** 1 Internal Medicine, University of Bahri, Khartoum, SDN; 2 Internal Medicine, Department of Medicine, Delma Hospital, Abu Dhabi, ARE; 3 Faculty of Medicine, Elrazi University, Khartoum, SDN; 4 Internal Medicine, Sudan Medical Specialization Board, Khartoum, SDN; 5 Faculty of Medicine, University of Gezira, Wad Madani, SDN; 6 General Medicine, NMC Royal Hospital, Abu Dhabi, ARE; 7 General Practice, Meena Health Care, Riyadh, SAU; 8 Internal Medicine, Sharaf Hospital, Hail, SAU; 9 Internal Medicine, Hail General Hospital, Hail, SAU; 10 Family Medicine, Al Hada Armed Forces Hospital, Taif, SAU; 11 Internal Medicine, Saudi German Hospital, Madinah, SAU; 12 Medicne and Surgery, University of Gezira, Wad Madani, SDN; 13 General Practice, Bassam First Medical Company, Duba, SAU

**Keywords:** clinical audit, haemovigilance, transfusion safety, vital-sign monitoring, who guidelines

## Abstract

Background: Blood transfusion is a lifesaving intervention; however, preventable harm can occur when bedside identification, monitoring, and documentation are incomplete. We focused on these bedside steps because they are safety critical, reduce the risk of wrong patient transfusions, and support early recognition of transfusion reactions.

Objective: To assess compliance with World Health Organization (WHO)-aligned bedside transfusion safety and monitoring criteria and hemovigilance reporting at the Al-Kareemat Specialized Complex for Clinics and Inpatient Care in Sudan, and to evaluate changes after a targeted quality improvement intervention using a two-cycle closed-loop audit design. This audit evaluated compliance with WHO-aligned transfusion safety, monitoring, and hemovigilance protocols at the Al-Kareemat Specialized Complex for Clinics and Inpatient Care in Sudan.

Methods: A closed-loop, two-cycle documentation-based clinical audit was conducted. The baseline cycle retrospectively reviewed 220 transfusion episodes administered between January and June 2024 using a structured checklist assessing eight bedside safety and monitoring criteria and hemovigilance indicators; absent documentation was classified as noncompliance. Following implementation of a targeted quality improvement package, focused on staff reinforcement and standardized monitoring and reporting documentation, a one-year re-audit of 120 transfusion episodes administered between January and June 2025 was performed using the same criteria and abstraction approach.

Results: At baseline, patient identification was documented in 96.8% (n = 213/220) of episodes. Monitoring and completion documentation were suboptimal, with 15-minute observation documented in 69.5% (n = 153/220) and end-of-transfusion vital signs in 80.9% (n = 178/220). Suspected transfusion reactions were similar across cycles, occurring in 2.7% (n = 6/220) at baseline and 2.5% (n = 3/120) at re-audit; formal hemovigilance reporting improved from 33.3% (n = 2/6) of suspected reactions to 100.0% (n = 3/3). In the re-audit, 15-minute observation documentation increased to 90.0% (n = 108/120), end-of-transfusion vital signs increased to 91.7% (n = 110/120), and 100.0% (n = 3/3) of suspected reactions were officially reported.

Conclusion: While foundational pre-transfusion safety steps were strong, baseline deficits were evident in active monitoring and hemovigilance reporting. The quality improvement intervention was associated with substantial improvements sustained at one-year re-audit. Continued reinforcement of standardized documentation and periodic repeat audits are recommended to maintain progress and achieve full compliance with safety-critical standards.

## Introduction

Blood transfusion is a fundamental therapeutic intervention in managing conditions such as obstetric hemorrhage, trauma, severe anemia, and major surgical procedures. The World Health Organization (WHO) identifies the safe and appropriate clinical use of blood and blood products as a cornerstone of effective health systems [1]. Evidence from hemovigilance programs indicates that a substantial proportion of transfusion-related harm is linked to preventable issues at the point of care, and bedside procedures represent the final and often most frequent point of vulnerability, including patient misidentification, administration errors, delayed recognition of acute reactions, and inadequate monitoring [2].

WHO-aligned clinical guidance advocates a structured pre-transfusion safety check to verify the correct patient and blood component using at least two unique identifiers before administration [3]. In our setting, these checkpoints are especially critical because they are practical, high-impact steps that can be implemented consistently and verified through routine clinical documentation. Monitoring protocols recommend documenting baseline observations immediately before transfusion, performing and documenting close observation during the initial 15 minutes, and recording vital signs upon completion of the transfusion [3]. Patient blood management frameworks further underscore the importance of safe administration practices to minimize avoidable risks [4]. When a transfusion reaction is suspected, prompt documentation and formal reporting through established hemovigilance channels are essential to protect patients and support system-level learning [5].

To our knowledge, this is the first clinical audit from Sudan to evaluate transfusion safety monitoring and hemovigilance documentation using a two-cycle quality improvement approach. This audit was designed to quantify baseline compliance with safety-critical bedside steps, implement targeted improvement measures, and assess whether gains were sustained on re-audit.

In practice, safe transfusion administration depends on consistent staff adherence to these procedural steps, including accurate verification, timely monitoring, and complete documentation [6]. However, implementation gaps persist, particularly in resource-limited settings where structured audit cycles are not routinely integrated into clinical governance. A WHO African Region survey highlighted deficiencies in facility-level oversight of transfusion quality, including limited routine auditing and monitoring [7]. Published evidence remains limited, and we found no previous Sudanese audit that has formally assessed compliance with these guidelines, which underscores the novelty and significance of this clinical audit. In this context, clinical audit provides a pragmatic approach to quantify documentation gaps, implement targeted improvements, and reassess performance over time.

Objective

This audit assessed compliance with WHO-aligned bedside transfusion safety and monitoring criteria and hemovigilance reporting practices at Al-Kareemat Specialized Complex for Clinics & Inpatient Care, Sudan.

## Materials and methods

Study design and setting

This project was conducted as a closed-loop, two-cycle clinical audit of transfusion safety and monitoring documentation. Cycle 1 consisted of a retrospective baseline review of routinely completed transfusion records. A targeted quality improvement package was then implemented, followed by cycle 2, a re-audit using the same standards, operational definitions, and abstraction approach. The audit was conducted at Al-Kareemat Specialized Complex for Clinics and Inpatient Care, Sudan, a tertiary facility with a central blood bank. During both audit periods, transfusion monitoring and documentation were paper-based, and routine service delivery was affected by resource constraints, including limited staff-to-patient ratios and intermittent power outages, which could delay or reduce documentation completeness.

Audit periods

The baseline audit covered all eligible transfusion episodes administered between 1 January and 30 June 2024, totaling 220 episodes. After implementation of the improvement package, the re-audit covered all eligible transfusion episodes administered between 1 January and 30 June 2025. The re-audit applied the same compliance rules and data abstraction form used in the baseline cycle and included 120 eligible transfusion episodes. The 12-month interval was selected to assess sustained adoption of the intervention and to reduce the likelihood that observed improvements reflected only short-term post-training effects.

Audit population and unit of analysis

The unit of analysis was a single transfusion episode, defined as the administration of one unit of a blood component to one patient at a specific time. Episodes were identified from routine blood bank and ward documentation by (1) listing all issued blood component units from the blood bank issue register during the audit periods and (2) tracing each issued unit to the corresponding ward documentation and patient medical record for verification and abstraction. Using an episode-level unit enabled consistent, like-for-like comparison of documentation performance across patients and between the baseline and re-audit cycles.

Episodes were included if documentation confirmed that a transfusion was administered during the audit period and the record set contained at least one assessable bedside criterion on the audit tool. Episodes were excluded if (a) the transfusion could not be verified from available documentation, (b) the corresponding patient record and transfusion documents could not be located after tracing from the issue register, or (c) none of the audit criteria were assessable because the transfusion record set was absent or blank. Reasons for exclusion were logged during case-tracing to characterize missing or unavailable documentation.

Compliance with each criterion was determined solely by the presence or absence of the required documentation in the patient record and associated transfusion documents.

Audit standards, criteria, and targets

Audit standards were derived from WHO-aligned recommendations for bedside transfusion safety and monitoring. Eight bedside criteria were assessed in both audit cycles: documentation of patient identification, documentation of informed consent, documentation of pre-transfusion baseline vital signs, documentation of a 15-minute observation after transfusion initiation, documentation of end-of-transfusion vital signs and completion, completion within four hours or less, traceability, and staff signature and record completeness. Targets were set at 100% for safety-critical bedside checks that should be universally documented because they directly prevent wrong blood events and support timely recognition of acute transfusion reactions. Targets were set at 100% for clinical steps, except for informed consent, with a target of 90% or higher to allow for emergency exceptions, and hemovigilance reporting, with a target of 90% or higher for suspected reactions. The 90% threshold for consent and hemovigilance was used because justified exceptions and practical constraints can occur, particularly in urgent scenarios for consent and in suspected reaction reporting, where event rarity and documentation workflow can affect completeness, while still maintaining an appropriately high standard for quality improvement. Traceability was considered compliant when the unit identifier was documented in the patient record and/or the transfusion tag was returned, according to the local process. The four-hour criterion was operationalized as a documented transfusion duration of four hours or less, calculated from the recorded start time to the recorded end or completion time. When start time, end time, or completion time were missing, the episode was classified as non-compliant for this criterion under the documentation-based compliance rule.

Data sources and data collection

Data were extracted from routinely maintained clinical documents, including blood bank issue registers, compatibility testing records, transfusion request forms, nursing observation charts, and patient medical records. A structured audit checklist and abstraction form standardized data collection across both audit cycles, including bedside criteria and reaction reporting fields, as detailed in the Appendices. Two auditors were trained on the operational definitions and abstraction procedures before data collection. To support consistency, 22 baseline episodes, representing 10% of the baseline sample, underwent independent double review, and discrepancies were resolved by consensus with reference to the source documents.

Documentation-based compliance rule

Because the audit assessed routine practice using available written records, any required element that was not documented for an episode was classified as non-compliant for that criterion. This rule was applied consistently across both audit cycles to ensure comparability between baseline and re-audit estimates.

Suspected transfusion reaction and hemovigilance reporting

A suspected transfusion reaction was defined as any episode with documentation of new signs or symptoms occurring during the transfusion or within 24 hours of completion, recorded as a possible transfusion-related adverse event. Examples included fever or rigors, rash or urticaria, dyspnea, hypotension, back or chest pain, nausea or vomiting, or dark urine, when documented in temporal association with the transfusion. Formal hemovigilance reporting was defined as documented submission of a suspected transfusion reaction report using the facility reporting mechanism, or explicit documentation in the record that a report was filed. Reaction identification was documentation-based and was not intended as a formal clinical adjudication of reaction type.

Contextual staff feedback

Brief, unstructured feedback was collected from ward and blood bank staff during audit activities to contextualize documentation gaps and workflow barriers. Feedback was summarized descriptively to inform the improvement package and interpretation of findings, and was not treated as a formal qualitative component.

Improvement package

Following the baseline cycle, a targeted improvement package was implemented to address gaps in early monitoring, end-of-transfusion documentation, and hemovigilance reporting. The package had three linked components: (1) staff education on bedside transfusion safety and documentation standards; (2) competency checking using a skills verification checklist; and (3) workflow supports to prompt real-time documentation and reporting. Training content covered patient identification and traceability, baseline assessment and vital sign recording, 15-minute observation and escalation, end-of-transfusion documentation and time recording (including the ≤ four-hour target), and recognition and documentation of suspected transfusion reactions with the steps for hemovigilance reporting. Competency was documented using a standardized workshop record and skills verification checklist (Table 1).

**Table 1:** Transfusion safety training workshop record and skills verification checklist used in the improvement package at Al-Kareemat Specialized Complex for Clinics and Inpatient Care, Sudan. Standardized training workshop record and skills verification checklist used to document participant details, workshop logistics, and competency assessment for bedside transfusion safety, monitoring, traceability, and suspected reaction recognition and hemovigilance reporting.

Name	Ward or Unit	Signature	Pre-test	Post-test
Workshop title:		Date:		
Facility:		Department:		
Venue:		Duration:	Hours	
Facilitator(s):		Contact:		
Planned follow-up:	30-day mini audit	Workshop cycle:	Baseline □ Re-audit □	
Skills verification checklist				
Skill	Status			Assessor initials
Positive patient identification using two identifiers and wristband check	Achieved □ Needs retraining □			
					Baseline vital signs documented before the transfusion started	Achieved □ Needs retraining □			
Fifteen-minute observation documented	Achieved □ Needs retraining □								
					End-of-transfusion documentation completed	Achieved □ Needs retraining □			
Completion within four hours documented, with justification for exceptions	Achieved □ Needs retraining □			
					Traceability completed, unit number recorded, and tag return documented	Achieved □ Needs retraining □			
Suspected reaction recognition, immediate response, and hemovigilance reporting form completion	Achieved □ Needs retraining □								

Workflow supports included attaching a standardized bedside transfusion safety and monitoring checklist to patient observation charts, reinforcing the facility’s suspected transfusion reaction reporting mechanism (where to obtain the form and where/how to submit it), and placing unit-level reminders. A designated focal person in each participating area supported checklist availability, encouraged completion of key documentation steps, and acted as a contact point for questions during implementation. To support sustainability, refresher training will be scheduled to accommodate new staff and mitigate turnover, and the processes will be integrated into staff onboarding. Periodic compliance review with feedback will be undertaken to support continuous improvement and adherence to protocols over time.

Outcomes and analysis

Primary outcomes were the proportions of criterion-level compliance for each of the eight bedside criteria in each audit cycle. Hemovigilance performance was measured as the proportion of suspected transfusion reactions that were formally reported. Analyses were descriptive, and changes across cycles were summarized as absolute percentage point differences. Exploratory comparisons by ward and shift were undertaken where documentation permitted, and were interpreted as hypothesis-generating.

Ethical considerations

This was a clinical audit of routinely collected documentation conducted for quality improvement purposes. There was no direct patient contact, no alteration to clinical care, and no patient identifiers were recorded in the audit dataset. Data were handled confidentially in accordance with institutional policy. As a quality-improvement clinical audit using de-identified routinely collected records, formal IRB/ethics committee review was not required (or was waived under institutional policy), and individual patient consent was not applicable.

## Results

Audit sample and transfusion characteristics

A retrospective baseline audit (cycle 1) was conducted on 220 transfusion episodes administered between 1 January and 30 June 2024. Based on available blood bank records, all issued units with documented compatibility information were ABO- and RhD-compatible with the intended recipients. A follow-up re-audit (cycle 2) included all 120 eligible transfusion episodes administered between 1 January and 30 June 2025, using the same six-month time window and the same eligibility criteria, and these episodes were assessed using the same documentation-based compliance rule. Because this was a documentation-based audit, the absence of required documentation was classified as noncompliance for that criterion. All proportions are presented as percentages, with the corresponding numerator and denominator reported in brackets as % (n/N).

Compliance with WHO-aligned bedside safety criteria

At baseline, adherence to WHO-aligned bedside safety benchmarks varied across criteria. Compliance ranged from 96.8% (n = 213/220) for patient identification to 69.5% (n = 153/220) for documentation of the 15-minute observation. The most significant baseline gaps were observed in early monitoring and end-of-transfusion documentation, as well as in sub-target performance for informed consent documentation, completion within four hours, and traceability.

In the re-audit, compliance improved across all eight bedside criteria. Re-audit compliance ranged from 98.3% (n = 118/120) for patient identification to 90.0% (n = 108/120) for both 15-minute observation documentation and informed consent documentation. The most significant absolute improvements were observed in documentation of the 15-minute observation, informed consent, end-of-transfusion documentation, and completion within four hours. Despite marked gains, most safety-critical steps remained below the 100% target. Informed consent reached the predefined target of 90% or greater. Table 2 presents baseline and re-audit performance.

**Table 2:** Baseline (cycle 1) and re-audit (cycle 2) compliance with WHO-aligned bedside transfusion safety and monitoring criteria at Al-Kareemat Specialized Complex for Clinics and Inpatient Care. Values are presented as n/N (%), where n is the number of transfusion episodes meeting the criterion, and N is the total number of eligible episodes reviewed in each cycle (baseline, N = 220; re-audit, N = 120). Target indicates the predefined audit standard for each criterion. Change (percentage points) represents the absolute difference between re-audit and baseline compliance, expressed in percentage points. Compliance was assessed using a documentation-based rule, whereby missing documentation was classified as noncompliance.

Criterion	Target	Baseline, % (n= / )	Re-audit, % (n= / )	Change, percentage points
Patient identification	100%	96.8% (n=213/220)	98.3% (n=118/120)	1.5
Informed consent documented	90% or higher	78.2% (n=172/220)	90.0% (n=108/120)	11.8
Pre-transfusion baseline vital signs	100%	92.3% (n=203/220)	96.7% (n=116/120)	4.4
15-minute observation documented	100%	69.5% (n=153/220)	90.0% (n=108/120)	20.5
End-of-transfusion vital signs and completion	100%	80.9% (n=178/220)	91.7% (n=110/120)	10.8
Completion within 4 hours or less	100%	85.5% (n=188/220)	95.0% (n=114/120)	9.5
Traceability (unit number or tag return)	100%	89.5% (n=197/220)	96.7% (n=116/120)	7.2
Staff signature and record completeness	100%	90.9% (n=200/220)	95.8% (n=115/120)	4.9

Audit highlights

The most substantial improvement was documentation of the 15-minute observation, which increased by 20.5 percentage points, indicating better adherence to monitoring protocols during the highest-risk period of transfusion. The facility achieved the predefined target for informed consent documentation in the re-audit. Although all criteria improved, documentation of the 15-minute observation and end-of-transfusion vital signs remained approximately 10 percentage points below the 100% target.

Note on the four-hour criterion

Under the documentation-based audit rule, episodes without documented start and finish times were classified as noncompliant for transfusion timing. The observed gap may therefore reflect incomplete timing documentation, prolonged transfusion duration, or both.

Suspected transfusion reactions and hemovigilance reporting

At baseline, six suspected transfusion reactions were identified, representing 2.7% (n = 6/220) of episodes. Only two of these were formally reported to the hemovigilance system, representing 33.3% (n = 2/6) of suspected reactions. In the re-audit, three suspected transfusion reactions were identified, representing 2.5% (n = 3/120) of episodes, and all three were formally reported, representing 100.0% (n = 3/3), and meeting the predefined reporting target of 90% or greater. Table 3 summarizes hemovigilance performance.

**Table 3:** Hemovigilance performance at baseline (cycle 1) and re-audit (cycle 2). This table summarizes the frequency of suspected transfusion reactions and the proportion formally reported through the hemovigilance system. Values are presented as n/N (%). For suspected transfusion reactions, N is the total number of transfusion episodes reviewed in each cycle (baseline, N = 220; re-audit, N = 120). For reported to hemovigilance, N is the number of suspected transfusion reactions in that cycle (baseline, N = 6; re-audit, N = 3). Percentages are rounded to one decimal place. Change (percentage points) represents the absolute difference between re-audit and baseline compliance, expressed in percentage points (re-audit minus baseline). Minor differences may occur due to rounding.

Category	Baseline, % (n= / )	Re-audit, % (n= / )	Change, percentage points
Suspected transfusion reactions	2.7% (n=6/220)	2.5% (n=3/120)	-0.2
Reported to hemovigilance, among suspected reactions	33.3% (n=2/6)	100.0% (n=3/3)	66.7

Contextual barriers to reporting, summarized from unstructured staff feedback (response rate: n = /, ___%), suggested that night-shift workload, limited availability of reporting forms, and the perception that mild symptoms do not require escalation may contribute to under-reporting. To address these barriers, we recommend shift-specific reinforcement, including scheduled reminders or brief check-ins during night shifts to support consistent reporting. We also recommend improving the visibility and accessibility of reporting forms by placing them in high-use locations such as nurse stations and emergency trolleys to enable rapid reporting during busy periods.

Operational patterns

Where documentation permitted subgrouping, the exploratory review suggested lower compliance during high-workload periods, such as night shifts and in busier clinical areas. However, inconsistent recording of ward and shift variables across episodes limited robust comparative analysis.

## Discussion

Interpretation of findings

In the WHO African Region survey, documented gaps in blood safety systems and implementation capacity have been reported [7]. This underscores the value of closed-loop clinical audits to identify priority risks and track improvement over time. In this audit at Al-Kareemat Specialized Complex for Clinics and Inpatient Care, baseline compliance was relatively high for core pre-transfusion bedside steps, particularly patient identification and baseline observations. This suggests that foundational verification behaviors are reasonably embedded in routine practice, providing a strong platform for targeted improvement.

However, baseline documentation of monitoring during and immediately after transfusion did not consistently meet the target standard. The most substantial deficit was the missing documentation of the 15-minute observation. Monitoring guidance highlights the first 15 minutes as a critical window for early recognition of acute transfusion reactions and emphasizes timely observation and recording to support rapid escalation and safe continuity of care [8].

More broadly, evidence-based transfusion standards emphasize that consistent documentation is integral to safety, auditability, and institutional learning [6]. Where early monitoring is not documented, the clinical record becomes less reliable for post-event review and system improvement, even when bedside actions may have occurred.

Following implementation of the quality improvement package, improvements were observed across all eight bedside criteria in the re-audit. These included the largest absolute gain in 15-minute monitoring documentation, alongside improvements in consent recording, end-of-transfusion documentation, completion timing, and traceability. Consent documentation reached the predefined target in the re-audit, aligning with guidance that requires documenting the indication, benefits, risks, alternatives, and the patient’s agreement, with provisions for emergencies where deferred documentation is necessary [9]. In parallel, strengthening patient identification and traceability supports correct patient-product matching and reduces misidentification risk [10]. From a clinical safety perspective, better monitoring documentation supports earlier recognition and management of suspected transfusion reactions, which is central to safe transfusion care [8].

Although consent completion improved, consent quality depends on more than form completion. Professional guidance stresses that consent should be a documented process showing that meaningful information was shared and understood as far as possible [9]. Evidence also indicates that targeted education, structured documentation tools, and feedback loops can improve clinician compliance and the completeness of transfusion-related records [11]. Nevertheless, patient understanding of transfusion can remain variable, supporting the need for clear explanations and reliable documentation of the discussion, not only the signature [12]. These findings align with foundational WHO principles for safe transfusion practice, which emphasize standardized procedures, documentation, monitoring, and systems for identifying and learning from adverse events [3,5].

Despite marked improvements, several safety-critical criteria remained below a 100% target in the re-audit. This indicates residual gaps, particularly for time-dependent monitoring steps, and highlights the need for sustained supervision and routine feedback. Completion of transfusion within the recommended timeframe also improved, aligning with widely used practice standards intended to reduce risks associated with prolonged administration [3]. Under a documentation-based compliance rule, noncompliance may reflect incomplete recording, actual deviation, or justified exceptions that were not documented. Where deviations are clinically justified, documenting timing and rationale remains essential for safety and auditability [3].

Hemovigilance performance

Hemovigilance reporting was the weakest domain at baseline. Although suspected reactions were identified, only a minority were formally reported. Safety literature emphasizes that systems for recognizing, documenting, reviewing, and learning from transfusion-related events are key levers for reducing preventable harm and strengthening clinical governance [5]. In settings where governance structures and reporting pathways are not consistently visible at the ward level, reporting behavior may be reduced even when reactions are suspected. This limits the reliability of the transfusion system’s “safety signal,” because events may be detected clinically but not captured in a way that supports verification, trend monitoring, and system-level learning.

In the re audit, the incidence of suspected reactions was similar to baseline, while reporting improved markedly. Although the number of suspected reactions in cycle 2 was small, the direction of change supports the interpretation that clarifying escalation steps and reinforcing expectations can strengthen hemovigilance behaviors as part of routine care [5]. In practical terms, improved reporting increases confidence that suspected reactions are not only recognized but also documented and escalated through the formal pathway, which strengthens the reliability of reaction detection and the completeness of institutional reporting. Staff knowledge and competence are also known to influence transfusion safety practice, supporting continued education and reinforcement as a sustainability strategy [13].

Comparison with other studies

The baseline pattern of stronger pre-transfusion checks with weaker monitoring, consent documentation, and event reporting is consistent with findings reported in resource-constrained environments where systems, staffing pressures, and documentation tools may be variably implemented [7]. In particular, our largest baseline deficit and strongest improvement signal were concentrated in early monitoring documentation and hemovigilance reporting, which mirrors quality improvement literature showing that targeted standardization of high-risk bedside documentation steps can produce measurable gains in completeness and auditability. The improvements observed across cycles are also consistent with published quality improvement work showing that revising and standardizing documentation tools can measurably improve the completeness of transfusion request and process documentation [14]. Methodologically, standardized transfusion audit approaches have been implemented using WHO-aligned tools, including audits based on the WHO Basic Information Sheet. This supports the feasibility of applying standardized criteria to measure and improve practice [15].

Strengths and limitations

This audit evaluated real-world practice using multiple routine data sources and applied a consistent documentation-based compliance rule across both cycles, enhancing internal comparability. A key limitation is that documentation audits cannot distinguish actions not performed from actions performed but not recorded. Treating missing documentation as noncompliance is conservative for safety auditing, but it may underestimate actual bedside practice. Future cycles could incorporate periodic direct observation or structured staff self-audits to better differentiate true noncompliance from missing documentation. Baseline and re-audit sample sizes differed because all eligible episodes were included in each period, and staffing and workload factors were not systematically measured.

Additionally, performance may have been influenced by a Hawthorne effect, since staff awareness of auditing can temporarily improve documentation and behavior. This audit did not include a concurrent control group, so improvements cannot be attributed to the intervention with certainty. Although the re-audit demonstrates improvement at one year, sustainability beyond this period remains uncertain and will require ongoing monitoring through future cycles. Finally, findings may have limited generalizability because this was a single-center audit in one facility, and local workflow, staffing, and documentation systems may differ across settings.

Recommendations and quality improvement actions

Given that the most significant baseline gaps were in early monitoring documentation and hemovigilance reporting, sustained efforts should prioritize these domains. Dedicated fields for baseline, 15-minute, and end-of-transfusion checks should be permanently embedded within routine observation charts. Reaction reporting forms and clear escalation steps should be consistently available and easily accessible across all clinical areas. Ward-based transfusion safety focal persons can provide peer support, reinforcement, and oversight of documentation completion. Regular data-driven feedback should be provided to clinical teams using the audit criteria as performance indicators. A third audit cycle should be planned to confirm the durability of improvements and to track progress toward full compliance with safety-critical transfusion standards.

Finally, strengthening standardized documentation and reporting supports broader learning and benchmarking. Global evaluations of blood safety data systems emphasize that usable, standardized datasets are necessary for tracking performance, learning from adverse events, and sustaining improvements at scale [16].

## Conclusions

At Al-Kareemat Specialized Complex for Clinics and Inpatient Care, baseline compliance with essential pre-transfusion checks was high. However, important gaps were identified in early monitoring documentation, end-of-transfusion documentation, consent recording, procedural timing documentation, and hemovigilance reporting. Following a targeted quality improvement intervention, the re-audit demonstrated marked improvement across all bedside criteria, particularly in 15-minute monitoring documentation, and achieved complete reporting of all suspected transfusion reactions. Continued reinforcement of these protocols and a subsequent audit cycle are recommended to sustain gains and progress toward full compliance with safety-critical transfusion standards.

## Appendices

**Table 4:** Audit and re-audit forms. Clinical Audit of Transfusion Safety and Monitoring at Al-Kareemat Specialized Complex for Clinics & Inpatient Care, Sudan. These appendices are designed for printing and routine use. All checkboxes use the symbol □.

Criterion	Assessment	Evidence source (e.g., chart and form)
1. Patient identification (2 IDs + wristband checked against unit label)	☐ Compliant ☐ Non-compliant ☐ N/A	
2. Informed consent (including emergency deferred documentation)	☐ Compliant ☐ Non-compliant ☐ N/A	
3. Baseline vital signs (documented before transfusion start)	☐ Compliant ☐ Non-compliant ☐ N/A	
4. 15-minute observation (vitals documented at 15-min mark)	☐ Compliant ☐ Non-compliant ☐ N/A	
5. End of transfusion (vitals and completion time documented)	☐ Compliant ☐ Non-compliant ☐ N/A	
6. Traceability (unit # recorded and tag return documented)	☐ Compliant ☐ Non-compliant ☐ N/A	
7. Record completeness (legible staff signature and all fields filled)	☐ Compliant ☐ Non-compliant ☐ N/A	

Reaction reporting

Suspected transfusion reaction? ☐ No ☐ Yes

If YES, reported to Hemovigilance? ☐ Yes ☐ No ☐ N/A

Evidence in notes/form: ________________________________________________

Date/Time Reported: ____ / ____ / ______ Time: ____ : ____

IV. AUDITOR SIGN-OFF

Auditor Initials: ________ Date: ____ / ____ / ______

Comments:

Additional comments:
